# Predictors of Cardiac Rehabilitation Utilization in England: Results From the National Audit

**DOI:** 10.1161/JAHA.116.003903

**Published:** 2016-10-21

**Authors:** Jennifer Sumner, Sherry L. Grace, Patrick Doherty

**Affiliations:** ^1^ Department of Health SciencesUniversity of YorkUK; ^2^ School of Kinesiology and Health ScienceFaculty of HealthYork UniversityTorontoCanada; ^3^ University Health NetworkUniversity of TorontoCanada

**Keywords:** cardiac, cardiac rehabilitation, patient compliance, patient factors, uptake, utilization, Health Services, Mortality/Survival

## Abstract

**Background:**

Cardiac rehabilitation (CR) is grossly underused, with major inequities in access. However, use of CR and predictors of initiation in England where CR contracting is available is unknown. The aims were (1) to investigate CR utilization rates in England, and (2) to determine sociodemographic and clinical factors associated with CR initiation including social deprivation.

**Methods and Results:**

Data from the National Audit of CR, between January 2012 and November 2015, were used. Utilization rates overall and by deprivation quintile were derived. Logistic regression was performed to identify predictors of initiation among enrollees, using the Huber–White–sandwich estimator robust standard errors method to account for the nested nature of the data. Of the 234 736 (81.5%) patients referred to CR, 141 648 enrolled, 97 406 initiated CR, and of those initiating, 37.2% completed a program of ≥8 weeks duration. The significant characteristics associated with CR initiation were younger age (odds ratio [OR] 0.98, 95% CI 0.98–0.99), having a partner (OR 1.31, 95% CI 1.17–1.48), not being employed (OR 0.86, 95% CI 0.77–0.96), not having diabetes mellitus (OR 0.84, 95% CI 0.77–0.92), greater anxiety (OR 1.02, 95% CI 1.003–1.04), not being a medically managed myocardial infarction patient (OR 0.57, 95% CI 0.42–0.76), and having had coronary artery bypass graft surgery (OR 1.64, 95% CI 1.09–2.47).

**Conclusions:**

CR enrollment does not meet English National Health Service targets; however it compares with that in other countries. Evidence‐based approaches increasing CR enrollment and initiation should be applied, focusing on the identified characteristics associated with CR initiation, specifically older, single, employed individuals with diabetes mellitus and those not revascularized.

## Introduction

Cardiac rehabilitation (CR) is an outpatient chronic disease management program designed to optimize secondary prevention and improve quality of life.^1^, ^2^ Participation in CR is associated with reduced cardiovascular mortality and hospital readmission among other benefits.^1^, ^2^ Accordingly, patients in the United Kingdom and several other countries have access to preventative CR programs. However, when viewed from a global perspective, CR is grossly underused. Recent meta‐analyses showed that in the last decade ≈43% of patients are referred,^3^ 40% enroll,^4^ and those who initiate CR adhere to an average of 67% of prescribed sessions.^4^ Greater participation is associated with lower mortality in a dose–response fashion,^5^ and hence it is imperative that CR utilization be increased to optimize outcomes at the population level.

There has been considerable research undertaken, both qualitative and quantitative, to understand factors associated with insufficient patient utilization of CR. A meta‐synthesis of qualitative studies suggested that patients' knowledge of CR services, perceptions of cardiovascular disease, as well as financial and occupational constraints are key factors influencing their utilization.^6^ Data from several registries in the United States and Europe have quantified sociodemographic and clinical characteristics associated with utilization. For example, data from 780 patients in the American Heart Association Get with the Guidelines database showed that nonwhite patients were much less likely to enroll than their white counterparts.^7^ Data from 2096 myocardial infarction (MI) patients in the Prospective Registry Evaluating outcomes after MI showed that women, patients with hypertension or peripheral artery disease, and those without health insurance were less likely to participate 1 month postdischarge. Furthermore, older, nonwhite, smokers, and those of less economic means and educational attainment were significantly less likely to participate 6 months post discharge. Patients who had a percutaneous coronary intervention (PCI) were less likely to participate at either time‐point.^8^ In Europe, data from the EUROASPIRE III survey of 13 935 patients showed older, female patients who did not have coronary artery bypass graft (CABG) surgery and those who smoked were less likely to attend.^9^ Consistent with the above findings regarding the centrality of financial/socioeconomic factors, numerous studies have also demonstrated social deprivation (eg, income, employment, and education) as a key factor associated with both low CR utilization and higher mortality.^10^, ^11^, ^12^

To date, research on the determinants of CR initiation in English cohorts has been limited, and stems only from small nonrepresentative samples.^13^, ^14^, ^15^, ^16^ A more thorough investigation is required to identify country‐specific influencing factors that could inform targeted interventions to increase CR utilization. Accordingly, the aims of this study were to (1) investigate CR utilization rates in England, and (2) determine sociodemographic and clinical characteristics associated with CR initiation including social deprivation.

## Methods

This study is reported following the guidelines: Strengthening the Reporting of Observational Studies in Epidemiology (STROBE).

### Design and Data Source

The National Audit of CR (NACR), funded by the British Heart Foundation, is a web‐based registry of CR in England, Wales, and Ireland. Information on service delivery, utilization, as well as patient characteristics and outcomes is collected.^17^ Data are entered onto NACR by practitioners involved in CR delivery, according to a data dictionary (http://www.cardiacrehabilitation.org.uk/nacr/downloads.htm). Data on patients eligible for CR and those referred are entered onto NACR. Participation in NACR is high: in 2015 a total of 204/308 (66.2%) programs provided data to the NACR, in England alone 164 programs.^17^ Data were extracted retrospectively for this observational study.

At centers involved in NACR, CR‐indicated patients are typically approached by the CR team. Referral to a CR program is generally completed while patients are still in the hospital or shortly after discharge by phone for day case PCI patients. For agreeing patients, a pre‐CR assessment takes place, during which sociodemographic and clinical characteristics are recorded as well as attendance and outcome following CR. Across the United Kingdom, CR is delivered in accordance with the British Association of Cardiovascular Prevention and Rehabilitation's standards.^18^ This includes both center and home‐based self‐management approaches such as the Heart Manual.^19^, ^20^ Patients in the center‐based programs are typically offered 16 sessions over 8 weeks at a minimum.^1^

### Ethics

The NACR, through the Health and Social Care Information Centre, has approval from the Health Research Authority's Confidentiality Advisory Group (under Section 251 of the NHS Act 2006) to collect patient‐identifiable data without explicit consent from individual patients for the purposes of audit and research. Approval is reviewed annually. Separate ethical approval was therefore not required as part of this project.

### Measures

CR utilization was operationalized as referral, enrollment, initiation, and completion. CR referral was defined as completion of a written/fax or electronic/systematic referral form with receipt at the CR program. CR enrollment was defined as attendance at the pre‐CR assessment. The dependent variable of CR initiation was defined as commencement of CR following the pre‐CR assessment (ie, initiate the exercise program, for at least 1 session). Patients were defined as CR‐initiators and noninitiators accordingly. Finally, CR completion was defined as receiving CR for ≥8 weeks, as per UK minimum standards.^1^ This was confirmed where participants had a program end date and/or post‐CR assessment entered at least 8 weeks from program initiation.

Sociodemographic characteristics assessed were age (years), sex (male/female), marital status (partnered/single), work status (employed/unemployed/retired), and ethnocultural background (White‐British, Asian, Other). Clinical characteristics included main referral indications: post‐MI (with medication management only), elective PCI, MI with PCI and CABG, prior cardiac history/event (yes/no), comorbidities including diabetes mellitus, risk factors (hypertension, physical inactivity, obesity as assessed via body mass index), as well as anxiety and depression symptoms. The latter were assessed on the Hospital Anxiety and Depression Scale (HADS), a reliable and well‐validated scale, with higher scores representing worse symptoms.^21^ Wait times were also calculated based on date of initiating event, referral date, enrollment date, and CR start date.

Finally, to investigate the impact of social deprivation on CR utilization, data from the 2015 English Indices of Deprivation, specifically the Index of Multiple Deprivation (IMD) reported at the Clinical Commissioning Group (CCG) level, were linked to NACR. Individual patients were assigned an IMD score according to the CCG in which their general practitioner was located. CCGs are clinically led bodies responsible for the planning and commissioning of healthcare services for their local area.

The IMD scores are based on 8 distinct domains of deprivation: income, employment, education, skills and training, health and disability, crime, barriers to housing and services, and living environment. These are combined, using appropriate weights, to calculate the IMD.^22^ For this study, IMD score was grouped into 5 equal‐sized groups according to score. Quintile 1 represents most‐deprived patients and quintile 5 represents least‐deprived patients. In some instances, individual patient general practitioner postal code was unavailable; thus CCG‐IMD could not be assigned.

### Participants

To test the first objective, all adult (≥18 years) cardiac patients in England entered onto the NACR between January 1, 2012 to November 5, 2015 were included. The main referral indications MI, MI with PCI, PCI, and CABG are presented separately; other indications such as heart failure were grouped in an “other” category. There were no exclusion criteria. For the second objective examining variables associated with CR initiation, only patients who attended the pre‐CR (enrolled) assessment were included, so data collected on their sociodemographic and clinical characteristics at that time were available. Data were restricted to those that had an IMD social deprivation score as well.

### Statistical Analysis

All analyses were conducted using STATA version 13.1. Descriptive statistics were used to describe CR utilization, and compare characteristics of CR initiators and noninitiators. Differences in these characteristics were then compared by initiation status using *t* tests, χ^2^, or Wilcoxon rank‐sum tests as appropriate. For continuous variables, standardized differences were also calculated to determine the meaningfulness of group differences irrespective of sample size. Differences greater than 0.1 were considered meaningful.^23^

A multivariate logistic regression was computed to assess factors associated with CR initiation. Variables were chosen for the multivariate analysis based on existing evidence indicating an association with initiation.^6^, ^7^, ^8^, ^9^, ^10^, ^11^, ^12^ Independent variables were age, sex, ethnicity, marital status, IMD quintile, employment status, comorbidity count, prior cardiac event, diagnosis of diabetes mellitus, anxiety and depressive symptoms, risk factors, and referral indication. To take account of the nested nature of the data (ie, patients treated within CR centers), the Huber–White–sandwich estimator robust standard errors method was used.

## Results

### Cohort Characteristics

**Figure 1:**
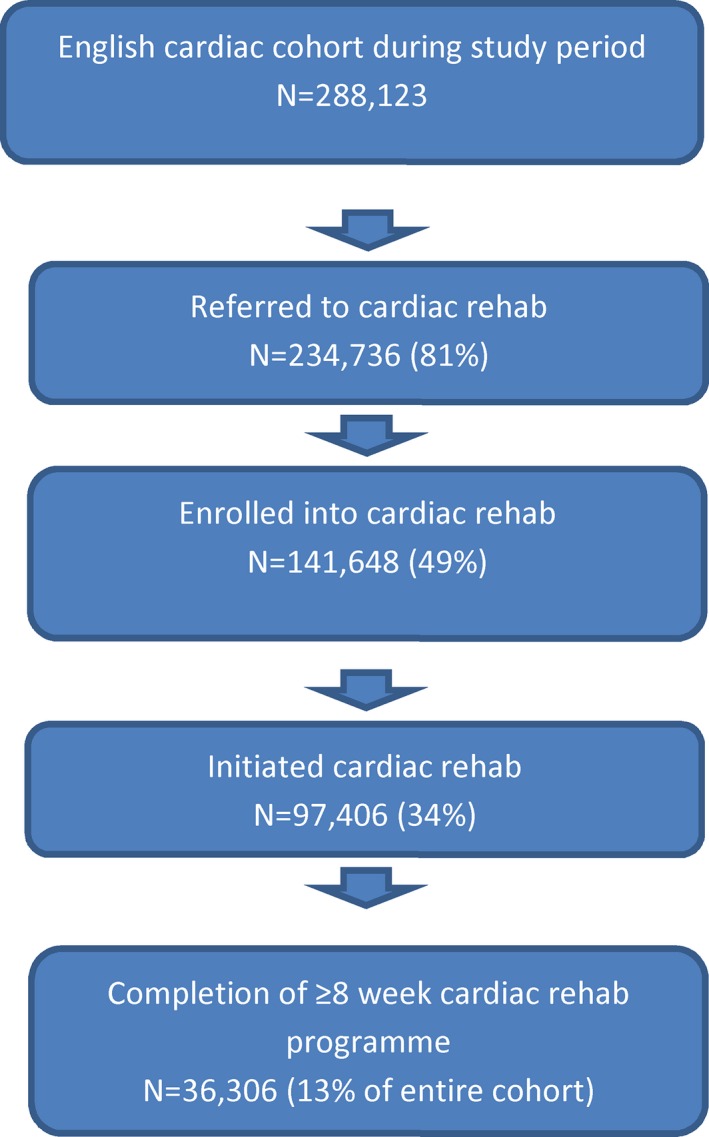
Patient flow in NACR and cardiac rehabilitation utilization. NACR indicates National Audit of Cardiac Rehabilitation.

As shown in Figure 1, the English NACR cohort comprised almost 300 000 patients during the period of study. A total of 98 880 referred English patients completed a pre‐CR assessment in the period of study (ie, enrolled) and had available deprivation data. Their characteristics are presented in Table 1. As shown, patients were primarily British, partnered, retired, males, had a comorbid condition, and were physically inactive. Other ethnocultural backgrounds were primarily black, Chinese, and those identifying as bi‐ and multiracial. Other CR referral indications were heart failure, valve surgery, implantable cardioverter‐defibrillator, and pacemakers. The most common cardiac history included MI, angina, and PCI.

**Table 1:** Sociodemographic and Clinical Characteristics of CR Initiators and Noninitiators

Characteristics	Overall N=98 880	CR Initiators n=55 953 (56.6%)	Noninitiators n=42 927 (43.4%)	*P* Value
Sociodemographic				
Mean age (SD)	65.79 (12.36)	64.53 (11.69)	67.43 (12.99)^a^	<0.001
Sex, n males	69 516 (72.0%)	40 510 (74.1%)	29 006 (69.2%)	<0.001
Ethnicity, n British				<0.001
White, British	69 095 (90.4%)	29 325 (91%)	39 770 (90%)	
Asian	5231 (6.8%)	2082 (6%)	3149 (7%)	
Other	2066 (2.7%)	808 (3%)	1258 (3%)	
Marital status, n partnered	55 282 (74.7%)	32 908 (77.5%)	22 374 (70.8%)	<0.001
Employment status, n				<0.001
Unemployed	9887 (15.9%)	6400 (16.4%)	3487 (15.1%)	
Employed	16 991 (27.4%)	11 405 (29.3%)	5586 (24.3%)	
Retired	35 022 (56.5%)	21 114 (54.2%)	13 908 (60.5%)	
English indices of deprivation quintile				<0.001
1 (most deprived)	14 269 (14.4%)	7749 (13.8%)	6520 (15.1%)	
2	18 431 (18.6%)	9190 (16.4%)	9241 (21.5%)	
3	16 048 (16.2%)	8562 (15.3%)	7486 (17.4%)	
4	25 070 (25.3%)	15 519 (27.7%)	9551 (22.2%)	
5 (least deprived)	25 062 (25.3%)	14 933 (26.6%)	10 129 (23.6%)	
Clinical				
Referral indication				
Post‐MI	16 910 (17.2%)	6985 (12.5%)	9925 (23.3%)	<0.001
MI‐PCI	30 552 (31.1%)	18 386 (33.0%)	12 166 (28.6%)	<0.001
PCI	17 783 (18.1%)	10 061 (18.1%)	7722 (18.1%)	0.824
CABG	15 110 (15.4%)	10 290 (18.5%)	4820 (11.3%)	<0.001
Other	17 756 (18.2%)	9859 (17.9%)	7897 (18.7%)	0.001
Comorbidity present (≥1)	65 560 (66.3%)	38 583 (68.9%)	26 977 (62.8%)	<0.001
Diabetic	15 928 (16.1%)	8876 (15.8%)	7052 (16.4%)	0.017
Prior cardiac event or procedure	32 896 (33.2%)	19 518 (34.8%)	13 378 (31.1%)	<0.001
Smoker	10 004 (21.3%)	4989 (17.2%)	5015 (27.9%)	<0.001
Physically inactive (<150 minutes per week)	60 346 (77.8%)	33 773 (73.7%)	26 573 (83.8%)	<0.001
Obese (BMI >30)	18 147 (29.6%)	11 814 (29.2%)	6333 (30.4%)	<0.001
Hypertensive (BP >140/90 mm Hg)	21 934 (32.1%)	13 763 (32.2%)	8171 (32.0%)	0.617
Mean Anxiety Score (SD)	5.73 (4.24)	5.78 (4.19)	5.61 (4.34)	<0.001
Mean Depression Score (SD)	4.61 (3.77)	4.60 (3.73)	4.62 (3.85)	0.286
Median time between initiating event and referral to CR, days^b^	4	4	3	
Median time between initiating event to prerehab assessment, days^b^	25	33	13	
Median time between referral and CR start, days	—	43	—	

Percentages were calculated using the denominator corresponding to the number of patients for which the characteristic was reported. BMI indicates body mass index; BP, blood pressure; CABG, coronary artery bypass graft surgery; CR, cardiac rehabilitation; MI, myocardial infarction; PCI, percutaneous coronary intervention; SD, standard deviation.

a Standardized difference >0.1.

b Capped at 365 days.

### CR Utilization

With regard to objective 1, CR utilization rates are shown in Figure 1. Over 80% of the cohort was referred to CR, 49.1% enrolled (attended pre‐CR assessment), and 33.8% initiated CR. Of those who initiated CR, 37.2% completed a program of at least 8 weeks duration. The mean program duration was 9.2 weeks or 65 days (SD 37.4; median=56 days). Wait times are shown in Table 1. Figures did not differ significantly between those with or without deprivation data (data not shown).

**Figure 2:**
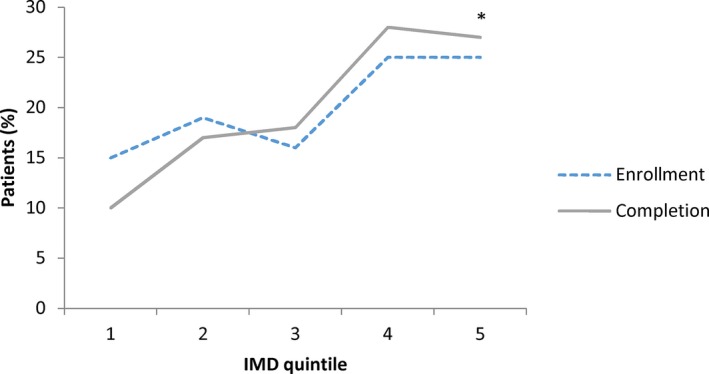
Proportion of patients (%) enrolled and completing CR by IMD quintile. CR indicates cardiac rehabilitation; IMD, Index of Multiple Deprivation. *Enrollment and completion compared in least (IMD quintile 1) vs most (IMD quintile 5) deprived group using χ^2^. For both tests, *P*<0.001.

As shown in Figure 2, there was a gradient in CR utilization based on degree of social deprivation. For each, those with lesser deprivation utilized CR to a greater degree (*P*<0.001).

### CR Initiators Versus Noninitiators

As shown in Table 1, 55 953 (56.6%) patients initiated CR following the pre‐CR assessment. A number of significant differences in participant characteristics were observed between CR initiators and noninitiators at a bivariate level. With regard to sociodemographic characteristics, noninitiators were significantly older, more often female, non‐British, single, retired, and at increased socioeconomic deprivation than CR initiators. With regard to clinical characteristics, noninitiators were more likely to have a referral indication of MI but less likely to have an indication of MI with PCI and CABG. Moreover, noninitiators had fewer comorbidities, less often had a prior cardiac event, were more physically inactive, and were more likely to be smokers than CR initiators. No meaningful differences in hypertension, anxiety or depressive symptoms, or wait times were observed. The association of age with CR initiation was particularly robust; for no other continuous variables was the standardized difference >0.1.

### Predictors of CR Initiation

Table 2 presents the findings from multivariate analysis. Smoking was not included in the model due to a high degree of missing data. The significant sociodemographic characteristics associated with initiation were the following: younger age, having a partner, and unemployment. The significant clinical characteristics associated with initiation were the following: not having diabetes mellitus, greater anxiety, not having a referral indication of MI without revascularization, and CABG surgery.

**Table 2:** Predictors of CR Initiation From Multivariate Regression

Variable	OR	95% CI	Significance (*P* Value)
Age	0.98	0.98 to 0.99	<0.001
Sex: female	0.96	0.89 to 1.03	0.294
Ethnicity (white, British as reference)			
Asian	1.36	0.91 to 2.05	0.127
Other ethnic groups	1.69	0.95 to 2.99	0.070
Marital status: Partnered	1.31	1.17 to 1.48	<0.001
IMD (group 3 reference)			
Quintile group 1	1.07	0.40 to 2.81	0.886
Quintile group 2	0.74	0.40 to 1.34	0.323
Quintile group 4	1.61	0.98 to 2.62	0.050
Quintile group 5	1.21	0.61 to 2.41	0.574
Employment status (retired as reference)			
Employed	0.86	0.77 to 0.96	0.011
Unemployed	0.95	0.80 to 1.13	0.627
≥1 Comorbidity	1.07	0.72 to 1.60	0.716
Prior cardiac event	0.87	0.73 to 1.04	0.147
Diabetic	0.84	0.77 to 0.92	<0.001
Anxiety score	1.02	1.003 to 1.04	0.017
Depression score	0.98	0.96 to 1.004	0.141
Physical inactivity	1.14	0.86 to 1.52	0.328
BMI	0.99	0.98 to 1.005	0.320
Blood pressure	0.98	0.82 to 1.16	0.831
Referral indication (other as reference)			
Post‐MI	0.57	0.42 to 0.76	<0.001
MI‐PCI	0.91	0.71 to 1.15	0.434
PCI	0.85	0.69 to 1.04	0.133
CABG surgery	1.64	1.09 to 2.47	0.017

BMI indicates body mass index; CABG, coronary artery bypass graft; CR, cardiac rehabilitation; IMD, Index of Multiple Deprivation; MI, myocardial infarction; OR, odds ratio; PCI, percutaneous coronary intervention.

## Discussion

To our knowledge, this is the largest cohort of patients in which CR utilization and predictors of CR initiation have been described. Generally CR was found to be underutilized. Factors associated with failure to initiate CR were generally consistent with what has been observed in other countries, namely, increasing age, nonpartnered status, less invasive treatment type, and the presence of comorbid diabetes mellitus;^7^, ^8^, ^9^, ^24^, ^25^, ^26^, ^27^ hence efforts should focus on enrolling these patient groups.

The average CR enrollment rates globally are 40%^4^; comparatively enrollment (defined as attendance at pre‐CR assessment) was found to be 50% in this study, with 34% of the cohort starting a CR program. While these rates are comparable, this is, however, still far from the target of 65% enrollment set by the National Health Service England^28^ and other clinical associations.^29^, ^30^, ^31^, ^32^ The level of completion in those who initiate CR is also worryingly low at 37.2%, and more work is needed to understand the reasons for this.

In terms of predictors of CR initiation, sociodemographic factors were partially consistent with work from other cohorts, although some differences were observed. Older age is consistently reported as a determinant of nonutilization,^24^, ^25^, ^26^, ^27^, ^33^ a finding reflected in this study. This is often attributed to lower referral rates among older patients, despite the fact that older patients have been shown to benefit from CR.^34^ Similarly, being in a relationship is often associated with increased enrollment,^35^ likely due to social support. Moreover, sex was not found to be significantly associated with initiation, although evidence from a recent systematic review showed enrollment may be predicted by sex.^4^

Interestingly, the multidimensional index of social deprivation was not a significant predictor of CR initiation in the multivariate model; however, employment alone was. This suggests that particular aspects of socioeconomic deprivation are pertinent to CR use. The impact of work status is evidently complex, with some studies reporting that employed patients are more likely to attend,^26^ which is likely a function of their higher socioeconomic status; others have shown that work may compete with the time needed for CR session participation and may lead to dropout.^6^, ^14^ Finally, other studies suggest that retired patients are more likely to attend (which is likely a function of time availability).

In relation to the clinical factors associated with noninitiation, some were consistent with existing evidence.^24^, ^27^, ^35^, ^36^ For example, data from 6874 referred cardiac patients in the Wisconsin CR Outcomes Registry showed that patients who had undergone CABG surgery were significantly more likely to enroll than patients who had not.^24^ It is likely that patients with more intensive/invasive acute cardiac intervention perceive greater mortality risk, and hence subsequent motivation to reduce this risk via CR participation. Moreover, presence of diabetes mellitus has consistently been associated with lower rates of enrollment.^24^, ^33^ Patients with diabetes mellitus likely have lower self‐efficacy in managing their diseases, due to their long history of being unable to tackle the lifestyle risk factors that cause cardiovascular disease. In relation to mental health, depressive symptoms were not associated with CR initiation but a small effect was observed for symptoms of anxiety in this cohort. This could be due to the greater burden of anxiety observed in the cohort than depression.

### Health Service Implications

Interventions to improve utilization have been recently reviewed.^37^ Successful strategies to increase enrollment included structured nurse‐ or therapist‐led contacts, early CR assessment appointments after hospital discharge, and motivational letters. These approaches should in particular be targeted to older, unpartnered patients who are working, have comorbid diabetes mellitus, and do not have CABG as a referral indication. Successful strategies to increase participation were self‐monitoring, action planning, and tailored counseling.

### Limitations

This large, multicenter investigation retrospectively accessed routinely collected patient data from an established national audit of CR services. However, some caution is warranted in interpreting the findings. First, although CR programs are encouraged to provide complete patients records, it was expected that a proportion of patient data would be missing. As such, smoking status could not be considered in the multivariate analysis. Second, because not all indicated inpatients are approached and entered into NACR, the rate of referred patients reported herein is likely inflated. Thus, referral rates should be interpreted with caution. Yet even in this select group, the problem of low enrollment, participation, and completion persists.

## Conclusions

Although the enrollment rate of ≈50% observed in England is below the recommended 65% benchmark, comparatively England has utilization rates consistent with what is observed in other countries. Factors associated with CR initiation should be considered as flags for CR practitioners as part of patient identification processes and during pre‐CR assessment. Evidence‐based interventions to increase utilization in these patients need to be broadly applied, so that the beneficial impact of CR in reducing cardiovascular mortality and morbidity can be optimized across the country. It was also evident that work is needed to improve the proportion of enrolling patients completing the recommended duration of CR, which was low at 37.2%.

## Sources of Funding

National Audit of Cardiac Rehabilitation (NACR) is funded by the British Heart Foundation. This research was not supported by any grants.

## Disclosures

None.

## References

[1] NICE . MI‐Secondary Prevention Guideline 172. London, UK: NICE; 2013.

[2] Anderson L , Thompson D , Oldridge N , Zwisler A , Rees K , Martin N , Taylor R . Exercise‐based rehabilitation for coronary heart disease. Cochrane Database Syst Rev. 2016;CD001800. doi: 10.1002/14651858.CD001800.pub3.26730878

[3] Colella T , Gravely S , Marzolini S , Grace S , Francis J , Oh P , Scott L . Sex bias in referral of women to outpatient cardiac rehabilitation? A meta‐analysis. Eur J Prev Cardiol. 2014;22:423–441. 24474091 10.1177/2047487314520783

[4] Samayoa L , Grace SL , Gravely S , Scott LB , Marzolini S , Colella TJ . Sex differences in cardiac rehabilitation enrollment: a meta‐analysis. Can J Cardiol. 2014;30:793–800. 24726052 10.1016/j.cjca.2013.11.007

[5] Martin BJ , Hauer T , Arena R , Austford LD , Galbraith PD , Lewin AM , Knudtson ML , Ghali WA , Stone JA , Aggarwal SG . Cardiac rehabilitation attendance and outcomes in coronary artery disease patients. Circulation. 2012;126:677–687. 22777176 10.1161/CIRCULATIONAHA.111.066738

[6] Clark AM , King‐Shier KM , Thompson DR , Spaling MA , Duncan AS , Stone JA , Jaglal SB , Angus JE . A qualitative systematic review of influences on attendance at cardiac rehabilitation programs after referral. Am Heart J. 2012;164:835–845. 23194483 10.1016/j.ahj.2012.08.020

[7] Mazzini MJ , Stevens GR , Whalen D , Ozonoff A , Balady GJ . Effect of an American Heart Association Get With the Guidelines program‐based clinical pathway on referral and enrollment into cardiac rehabilitation after acute myocardial infarction. Am J Cardiol. 2008;101:1084–1087. 18394437 10.1016/j.amjcard.2007.11.063

[8] Parashar S , Spertus JA , Tang F , Bishop KL , Vaccarino V , Jackson CF , Boyden TF , Sperling L . Predictors of early and late enrollment in cardiac rehabilitation, among those referred, after acute myocardial infarction. Circulation. 2012;126:1587–1595. 22929302 10.1161/CIRCULATIONAHA.111.088799

[9] Kotseva K , Wood D , De Backer G , De Bacquer D . Use and effects of cardiac rehabilitation in patients with coronary heart disease: results from the EUROASPIRE III survey. Eur J Prev Cardiol. 2013;20:817–826. 22718794 10.1177/2047487312449591

[10] Barnard J , Grant SW , Hickey GL , Bridgewater B . Is social deprivation an independent predictor of outcomes following cardiac surgery? An analysis of 240,221 patients from a national registry. BMJ Open. 2015;5:1–10. 10.1136/bmjopen-2015-008287 PMC4486967 26124512

[11] Thorne K , Williams JG , Akbari A , Roberts SE . The impact of social deprivation on mortality following acute myocardial infarction, stroke or subarachnoid haemorrhage: a record linkage study. BMC Cardiovasc Disord. 2015;15:1–10. 26187051 10.1186/s12872-015-0045-x PMC4506594

[12] Gaalema D , Higgins S , Shepard D , Suaya J , Savage P , Ades P . State‐by‐state variations in cardiac rehabilitation participation are associated with educational attainment, income, and program availability. J Cardiopulm Rehabil Prev. 2014;34:248–254. 24820451 10.1097/HCR.0000000000000059 PMC4098712

[13] Melville MR , Packham C , Brown N , Weston C , Gray D . Cardiac rehabilitation: socially deprived patients are less likely to attend but patients ineligible for thrombolysis are less likely to be invited. Heart. 1999;82:373–377. 10455092 10.1136/hrt.82.3.373 PMC1729163

[14] McKee G , Biddle M , O' Donnell S , Mooney M , O' Brien F , Moser D . Cardiac rehabilitation after myocardial infarction: what influences patients' intentions to attend? Eur J Cardiovasc Nurs. 2014;13:329–337. 23818214 10.1177/1474515113496686

[15] Lane D , Carroll D , Ring C , Beevers DG , Lip GY . Predictors of attendance at cardiac rehabilitation after myocardial infarction. J Psychosom Res. 2001;51:497–501. 11602219 10.1016/s0022-3999(01)00225-2

[16] Bethell H , Lewin R , Evans J , Turner S , Allender S , Petersen S . Outpatient cardiac rehabilitation attendance in England: variability by region and clinical characteristics. J Cardiopulm Rehabil Prev. 2008;28:386–391. 19008693 10.1097/HCR.0b013e31818c3b44

[17] The National Audit of Cardiac Rehabilitation (NACR) . The National Audit of Cardiac Rehabilitation Annual Statistical Report. York, UK: University of York; 2015.

[18] Buckley JP , Furze G , Doherty P , Speck L , Connolly S , Hinton S , Jones JL . BACPR scientific statement: British standards and core components for cardiovascular disease prevention and rehabilitation. Heart. 2013;99:1069–1071. 23403407 10.1136/heartjnl-2012-303460

[19] Lewin B , Robertson I , Cay E , Irving J , Campbell M . Effects of self‐help post‐myocardial‐infarction rehabilitation on psychological adjustment and use of health services. Lancet. 1992;339:1036–1040. 1349062 10.1016/0140-6736(92)90547-g

[20] Dalal HD , Doherty P , Taylor RS . Cardiac rehabilitation. BMJ Open. 2015;351:h5000. 10.1136/bmj.h5000 PMC4586722 26419744

[21] Zigmond AS , Snaith RP . The hospital anxiety and depression scale. Acta Psychiatr Scand. 1983;67:361–370. 6880820 10.1111/j.1600-0447.1983.tb09716.x

[22] Government Department for Communities and Local Government . English Indices of Deprivation 2015 Statistical Report. London, UK: UK Government; 2015.

[23] Mamdani M , Sykora K , Li P , Normand SL , Streiner DL , Austin PC , Rochon PA , Anderson GM . Reader's guide to critical appraisal of cohort studies: 2. Assessing potential for confounding. BMJ. 2005;330:960–962. 15845982 10.1136/bmj.330.7497.960 PMC556348

[24] Turk‐Adawi KI , Oldridge NB , Tarima SS , Stason WB , Shepard DS . Cardiac rehabilitation enrollment among referred patients: patient and organizational factors. J Cardiopulm Rehabil Prev. 2014;34:114–122. 24142042 10.1097/HCR.0000000000000017

[25] Strens D , Colle A , Vrijens F , Paulus D , Eyssen M , Van Brabandt H , Van Vlaenderen I . Multidisciplinary outpatient rehabilitation following cardiac revascularization or valve surgery: patient‐related factors for uptake. Eur J Prev Cardiol. 2013;20:422–430. 22392164 10.1177/2047487312441727

[26] Cooper AF , Jackson G , Weinman J , Horne R . Factors associated with cardiac rehabilitation attendance: a systematic review of the literature. Clin Rehabil. 2002;16:541–552. 12194625 10.1191/0269215502cr524oa

[27] Chamosa S , Alarcon JA , Dorronsoro M , Madruga FJ , Barrera J , Arrazola X , de la Cuesta P , Alkiza ME , Begiristain JM , Carrera I , San Vicente JM . Predictors of enrollment in cardiac rehabilitation programs in Spain. J Cardiopulm Rehabil Prev. 2015;35:255–262. 26110624 10.1097/HCR.0000000000000126

[28] Department of Health . Cardiovascular Disease Outcomes Strategy. London, UK: Department of Health; 2013.

[29] Cardiac Network Wales . Cardiac Rehabilitation Review and Recommendations. Cardiff, UK: Cardiac Network; 2013.

[30] Scottish Intercollegiate Guidelines Network (SIGN) . Cardiac Rehabilitation Guideline no. 57. Cardiac Rehabilitation. Edinburgh, UK: SIGN; 2002.

[31] CREST . Guidelines for Cardiac Rehabilitation in Northern Ireland. Belfast, UK: CREST; 2006.

[32] Chan PS , Oetgen WJ , Buchanan D , Mitchell K , Fiocchi FF , Tang F , Jones PG , Breeding T , Thrutchley D , Rumsfeld JS , Spertus JA . Cardiac performance measure compliance in outpatients: the American College of Cardiology and National Cardiovascular Data Registry's PINNACLE (Practice Innovation And Clinical Excellence) program. J Am Coll Cardiol. 2010;56:8–14. 20620710 10.1016/j.jacc.2010.03.043 PMC2922046

[33] Weingarten M , Salz K , Thomas R , Squires R . Rates of enrollment for men and women referred to outpatient cardiac rehabilitation. J Cardiopulm Rehabil Prev. 2011;31:217–222. 21317800 10.1097/HCR.0b013e318207d2fa PMC3137685

[34] Lavie CJ , Milani RV . Benefits of cardiac rehabilitation and exercise training programs in elderly coronary patients. Am J Geriatr Cardiol. 2001;10:323–327. 11684916 10.1111/j.1076-7460.2001.00636.x

[35] Grace S , Gravely‐Witte S , Brual J , Monette G , Suskin N , Higginson L , Alter D , Stewart D . Contribution of patient and physician factors to cardiac rehabilitation enrollment: a prospective multilevel study. Eur J Cardiovasc Prev Rehabil. 2008;15:548–556. 18830085 10.1097/HJR.0b013e328305df05 PMC2927524

[36] Brady S , Purdham D , Oh P , Grace S . Clinical and sociodemographic correlates of referral for cardiac rehabilitation following cardiac revascularization in Ontario. Heart Lung. 2013;42:320–325. 23998380 10.1016/j.hrtlng.2013.07.001

[37] Karmali K , Davies P , Taylor F , Beswick A , Martin N , Ebrahim S . Promoting patient uptake and adherence in cardiac rehabilitation (review). Cochrane Libr. 2014;CD007131. doi: 10.1002/14651858.CD007131.pub3. 10.1002/14651858.CD007131.pub3 24963623

